# Comparative genomics of mitochondria in chlorarachniophyte algae: endosymbiotic gene transfer and organellar genome dynamics

**DOI:** 10.1038/srep21016

**Published:** 2016-02-18

**Authors:** Goro Tanifuji, John M. Archibald, Tetsuo Hashimoto

**Affiliations:** 1 Faculty of Life and Environmental Sciences, University of Tsukuba, Japan; 2 Department of Biochemistry and Molecular Biology, Dalhousie University, Canada; 3 Program in Integrated Microbial Biodiversity, Canadian Institute for Advanced Research, Toronto, Ontario Canada

## Abstract

Chlorarachniophyte algae possess four DNA-containing compartments per cell, the nucleus, mitochondrion, plastid and nucleomorph, the latter being a relic nucleus derived from a secondary endosymbiont. While the evolutionary dynamics of plastid and nucleomorph genomes have been investigated, a comparative investigation of mitochondrial genomes (mtDNAs) has not been carried out. We have sequenced the complete mtDNA of *Lotharella oceanica* and compared it to that of another chlorarachniophyte, *Bigelowiella natans*. The linear mtDNA of *L. oceanica* is 36.7 kbp in size and contains 35 protein genes, three rRNAs and 24 tRNAs. The codons GUG and UUG appear to be capable of acting as initiation codons in the chlorarachniophyte mtDNAs, in addition to AUG. Rpl16, rps4 and atp8 genes are missing in *L.oceanica* mtDNA, despite being present in *B. natans* mtDNA. We searched for, and found, mitochondrial rpl16 and rps4 genes with spliceosomal introns in the *L. oceanica* nuclear genome, indicating that mitochondrion-to-host-nucleus gene transfer occurred after the divergence of these two genera. Despite being of similar size and coding capacity, the level of synteny between *L. oceanica* and *B. natans* mtDNA is low, suggesting frequent rearrangements. Overall, our results suggest that chlorarachniophyte mtDNAs are more evolutionarily dynamic than their plastid counterparts.

Endosymbiosis has played an important role in the generation of eukaryotic cellular diversity. The evolution of the mitochondrion from an alpha-proteobacterial endosymbiont was particularly significant: all known eukaryotes possess mitochondria or mitochondrion-derived organelles^1^,^2^. Plastids (chloroplasts) also evolved from bacteria by endosymbiosis, an event that paved the way for the evolution of a vast array of aquatic and terrestrial eukaryotic phototrophs. On multiple occasions the so-called ‘primary’ plastids of red and green algae were engulfed by non-photosynthetic eukaryotes and retained as secondary plastids^3^,^4^. During the cellular integration of host and endosymbiont, extensive genome reorganization in the form of gene loss and endosymbiont-to-host gene transfer took place^5^,^6^. Various aspects of organelle genome evolution are nevertheless still poorly understood.

Chlorarachniophyte algae belong to the eukaryotic supergroup Rhizaria, and together with cryptophyte algae, are an important lineage for the study of secondary endosymbiosis. This is because they contain endosymbiont-derived nuclei, nucleomorphs^7^,^8^, unusual organelles that reside between the second and third plastid membranes in a space corresponding to the cytosol of the engulfed eukaryotic endosymbiont (i.e., the periplastidal compartment or PPC). Nucleomorphs are intriguing given that other secondarily photosynthetic organisms such as diatoms and haptophytes have completely lost their endosymbiont-derived nuclei. The plastids and nucleomorphs of chlorarachniophytes and cryptophytes are derived from green algal and red algal endosymbionts, respectively^8^. Despite their independent origins, the nucleomorph genomes of chlorarachniophytes and cryptophytes have similar architectures, with three linear chromosomes, ribosomal RNA (rRNA) operons at the chromosome ends and highly reduced genomes <1 megabase pairs (Mbp) in size^9^,^10^,^11^,^12^.

Comparative genomics of nucleomorph-bearing organisms has shed light on the impact of secondary endosymbiosis on genome and cell evolution. Curtis *et al.* (2012) sequenced the nuclear genomes of the model chlorarachniophyte *Bigelowiella natans* and the model cryptophyte *Guillardia theta*, showing that both algal nuclear genomes are “mosaics” of genes derived from both host and endosymbiont^13^. Nevertheless, while mtDNA-derived DNA fragments (NUMTs) have been found in the nuclear genomes of both organisms, recently transferred nucleomorph and plastid DNA fragments (NUNMs and NUPTs, respectively) have not been identified^13^,^14^. Plastid-to-nucleus and nucleomorph-to-nucleus DNA transfer thus appears to be rare in these cells. This observation provides a possible explanation for why nucleomorphs persist in chlorarachniophytes and cryptophytes, i.e., because opportunities for the transfer of essential nucleomorph genes to the host nucleus by endosymbiotic gene transfer (EGT) are infrequent^13^.

Comparative genomics of nucleomorphs has revealed that a similar set of house keeping genes are retained in both chlorarachniophytes and cryptophytes, despite the independent origins of these endosymbiotically derived organelles^8^,^15^,^16^,^17^,^18^,^19^,^20^,^21^. As well, recombination within and among nucleomorph chromosomes appears to be frequent. These results suggest that similar reductive forces have been acting upon the nucleomorph genomes during the course of evolution.

Multiple chlorarachniophyte and cryptophyte plastid genomes have been sequenced^21^,^22^,^23^,^24^,^25^,^26^. The plastid gene repertories and genome structures are nearly identical between examined genera within each of these lineages, with the exception of the non-photosynthetic cryptomonad, *Cryptomonas paramecium* (the *C. paramecium* plastid genome has lost numerous photosynthesis-related protein genes but nevertheless retains strong synteny with the genomes of phototrophic strains)^21^,^22^,^23^,^24^,^25^,^26^. Two mitochondrial genomes (mtDNAs) have been sequenced from cryptophytes, those of *Rhodomonas salina* and *Hemiselmis andersenii*^27^,^28^. Although their overall gene sets are very similar, four protein genes (rps1, atp4, tatA and sdh4) are missing in *H. andersenii*, despite being present in *R. salina* mtDNA. More than 30 instances of genome rearrangement are thought to have occurred since *R. salina* and *H. andersenii* diverged from one another, perhaps mediated by the presence of large repeat regions^28^. In sum, the mtDNA of cryptophytes appears to be much more dynamic than their plastid genomes.

Information on the dynamics of mitochondrial genome evolution in chlorarachniophytes is currently lacking. Pulsed-field gel electrophoresis studies by Gilson and colleagues suggested that chlorarachniophyte mtDNAs have a linear architecture^29^,^30^. However, while the *B. natans* mtDNA sequence is publicly available (NCBI accession HQ840955), a detailed analysis has not been carried out. We have sequenced and annotated the mtDNA of *Lotharella oceanica* and compared it to that of *B. natans*. The linear structure of *L. oceanica* mtDNA was verified by a combination of Southern hybridization and genome mapping methods. The level of synteny between the mitochondrial genomes of the two organisms is unexpectedly low, suggesting frequent rearrangement. Although the protein gene sets found in these two mtDNAs are similar, at least two protein genes were recently transferred to the nuclear genome in *L. oceanica*. We explore possible reasons for the observed differences in the evolution of mitochondrial, plastid and nucleomorph genomes in nucleomorph-bearing algae.

## Results and Discussion

### The mitochondrial genome of *Lotharella oceanica*

**Figure 1:**
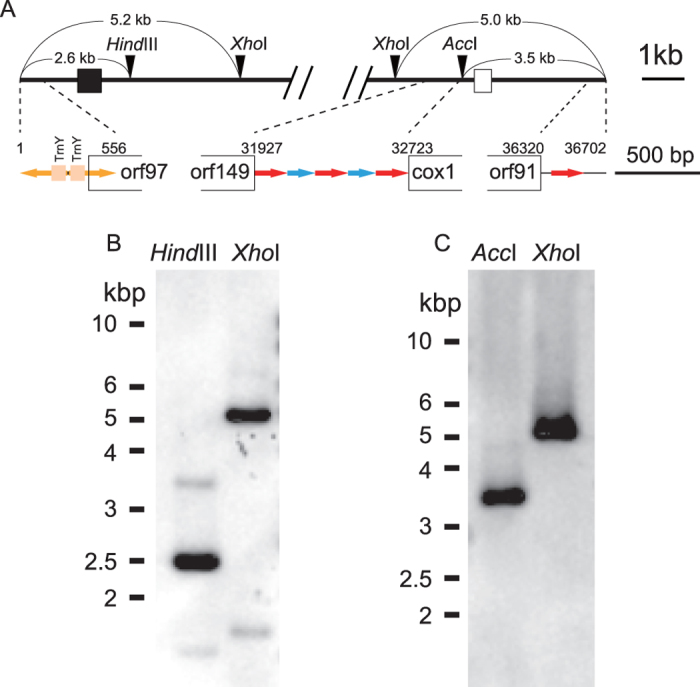
Physical map of the left and right termini of the *Lotharella oceanica* mitochondrial genome and Southern hybridization results. (**A**) Map of the left and right termini of the *Lotharella ocecanica* mitochondrial genome showing restriction enzyme sites (upper), and corresponding protein genes and repeated sequence regions (lower). Black and white boxes in the restriction enzyme sites map show the left and right terminus-specific probe regions, respectively. The arrows in yellow, red and blue indicate the type of repeated sequence. Numbers correspond to genome coordinates. (**B**) Southern hybridization images using the left terminus-specific probe with *Hind*III and *Xho*I restriction enzymes, and (**C**) the hybridization image using the right terminus-specific probe with *Acc*I and *Xho*I restriction enzymes. Bars on left sides of photos show the DNA size markers.

The mitochondrial genome (mtDNA) of the chlorarachniophyte *Lotharella oceanica* CCMP622 was sequenced and assembled. PCR and standard Sanger sequencing techniques were used to resolve inconsistencies in the consensus sequences obtained using different assembly methods and to fill gaps between scaffolds (see Material and methods). Genome heterogeneity was not observed during the course of our PCR experiments. A final contig 36,702 bp in length with ~800X Illumina sequence coverage was ultimately obtained. The genome contains one inverted repeat at position 1–556 and two types of tandem repeats (including 4 copies of a 196 bp element and two copies of a 167 bp repeat) (Fig. 1A). Inconsistencies between the different scaffolding methods and the sequencing gaps were presumably caused by these repetitive regions.

Mitochondrial genome architecture varies considerably across eukaryotic diversity^1^,^2^. In the case of chlorarachniophytes, linear mtDNA structures were suggested previously for six strains based on pulsed-field gel electrophoresis^29^,^30^. In *L. oceanica*, PCR experiments with ‘outward-facing’ primers corresponding to both termini of the mtDNA scaffolds failed to generate amplicons (data not shown), consistent with the existence of a linear structure for *L. oceanica* mtDNA as well. In order to verify a linear structure and search for the possible presence of additional terminal sequences in the *L. oceanica* mtDNA, a traditional genome mapping experiment was carried out using a combination of restriction enzyme digestions and Southern hybridization.

Figure 1A shows the predicted restriction enzyme cut sites and the locations of mtDNA terminus-specific probes used for Southern hybridization analysis. Based on the structure of our ‘final’ *L. oceanica* mtDNA genome assembly, restriction fragment sizes were predicted as shown. As expected, 2.6 and 5.2 kbp fragments were detected with *Hin*dIII and *Xho*I restriction enzyme digestions using the left-terminus specific probe (Fig. 1B), and 3.5 and 5.0 kbp fragments were detected with *Acc*I and *Xho*I using the right-terminus specific probe, respectively (Fig. 1C). These results confirm the linear structure of the *L. oceanica* mtDNA and robust nature of our genome assembly.

### Chlorarachniophyte mitochondrial genomes: structure, gene content, and initiation codon diversity

**Figure 2:**
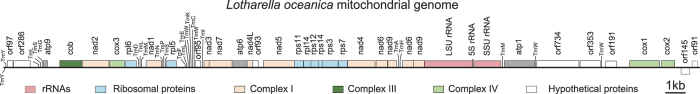
Physical map of the *Lotharella oceanica* mitochondrial genome. Colors correspond to predicted functional categories. Genes mapped on the upper side of the genome are transcribed right to left and those on the bottom, left to right.

The mtDNA of *Lotharella oceanica* contains 35 protein genes, three ribosomal RNA (rRNA) genes and 24 transfer RNA (tRNA) genes (Fig. 2, Tables 1 and 2). Nine of the 35 protein genes are hypothetical ORFs, encoding putative proteins that show no significant sequence similarity with known proteins including *B. natans* mitochondrial proteins (e-value < 1e-5). Three protein genes with predicted functions (atp8, rpl16 and rps4) and four tRNA genes (trnG (tcc), trnR (tcg), trnR (tct) and trnV (tac)) are missing in *L. oceanica* mtDNA, despite being present in *B. natans* mtDNA. The rpl16 and rps4 genes appear to have been transferred to the *L. oceanica* nuclear genome (see below). In addition, two copies of nad6, nad9, trnW (tca) and trnY (gta) are found in *L. oceanica* mtDNA, while single copies of these genes reside in *B. natans* mtDNA (Fig. 2). All genes with predicted functions in *L. oceanica* mtDNA, including RNA genes, can be found in *B. natans* mtDNA as well; the suite of genes found in the former is a subset of those in the latter (Table 2). The mtDNA-encoded proteins of both chlorarachniophytes are exclusively involved in oxidative phosphorylation (e.g., nad and cox proteins) and translation (e.g., rps and rpl proteins); components of general mitochondrial functions such as transcription, RNA processing, and protein import are completely lacking. No introns were found in the *L. oceanica* and *B. natans* mtDNAs.

The gene density of the *L. oceanica* mtDNA is 0.95 genes/kbp (73.2% coding), which is similar to that of *B. natans* (0.93 genes/kbp and 77.8% coding) (Table 1). The overall G + C content of *L. oceanica* mtDNA is 50.14%, higher than in *B. natans* (42.15%). Mitochondrial genomes and other reduced genomes such as those of plastids, nucleomorphs and endosymbiotic bacteria are often highly biased towards A + T^8^,^31^,^32^,^33^. For example, the G + C content of mitochondrial and plastid genomes are typically 30–40%^31^,^32^, lower than that of *L. oceanica* mtDNA. Since the ‘higher’ G + C is present in the protein coding regions as well (49.22% in *L. oceanica* and 40.81% in *B. natans*), the overall G + C content of *L. oceanica* mtDNA is not biased simply due to the presence of small regions of extreme G + C richness. Sequence data from a more diverse set of chlorarachniophyte mtDNAs will be necessary to determine whether the ‘high’ G + C content of the *L. oceanica* genome is an anomaly or a general feature of chlorarachniophyte mtDNAs.

A noteworthy feature of the chlorarachniophyte mtDNAs analyzed herein is the apparent use of GUG (Valine) and UUG (Leucine) as initiation codons, in addition to AUG (Methionine). In *L. oceanica*, the initiation codons of 12 of 35 protein genes (atp6, cob, cox2, cox3, nad5, nad7, nad9-1, nad9-2, rpl5, rps3, rps7 and rps14) are GUG (Valine), and three protein genes (orf97, orf191 and orf286) begin with UUG (Leucine). Out of 12 proteins with alternate initiation codons in *L. oceanica*, 11 have start codons in the exact same position as their homologs in *B. natans* and/or other organisms (methionine codons were not found within 20 amino acids upstream or downstream of any of the alternate initiation codons (V or L) in *L. oceanica*). In *B. natans*, the initiation codons of rps4 and atp8 are GUG (Valine), and that of orf91 is UUG (Leucine). These initiation codon usage patterns are similar to those in bacteria (i.e., AUG, GUG, UUG plus AUA). It is conceivable that the initiation codon usage patterns found in chlorarachniophyte mtDNAs were inherited directly from the alpha-proteobacteria progenitor of the mitochondrion. Additional mtDNA sequences, especially from other members of the Rhizaria, may shed light on this issue.

### Endosymbiotic gene transfer

Three protein genes (atp8, rps4 and rpl16) are absent from the *Lotharella oceanica* mitochondrial genome but present in *Bigelowiella natans* (Table 2). Given that these three proteins are generally necessary for energy production and translation in mitochondria, one explanation is that these three protein genes have moved to the *L. oceanica* nuclear genome by endosymbiotic gene transfer (EGT). In order to explore this possibility, a blastx search against the transcriptome and genome contigs was carried out with relaxed settings (e-value cut off = 0.1). Sequences corresponding to a partial rps4 gene and a full-length rpl16 gene were found in the transcriptome and genomic data. No atp8 candidate was found.

The sequence coverage depths of the genomic scaffolds on which the rps4 and rpl16 candidates were found are ~4X and ~5X, respectively, in stark contrast to that of mtDNA DNA (~800X). These observations are consistent with the idea that these sequences are present in the *L. oceanica* nuclear genome, especially given that the DNA sequenced was a CsCl-purified, organelle DNA-enriched sample^21^. The rps4 and rpl16 coding regions were both found to contain spliceosomal introns with GT-AG boundaries, removal of which is supported by alignments of transcriptome and genome data (Fig. S1). Furthermore, a mitochondrion-targeting signal was detected at the N-terminus of the mitochondrial rpl16 candidate by the subcellular localization prediction program TargetP^34^. Collectively, these data strongly suggest that the rps4 and rpl16 genes ‘recently’ migrated from the *L. oceanica* mtDNA to the nuclear genome.

**Figure 3:**
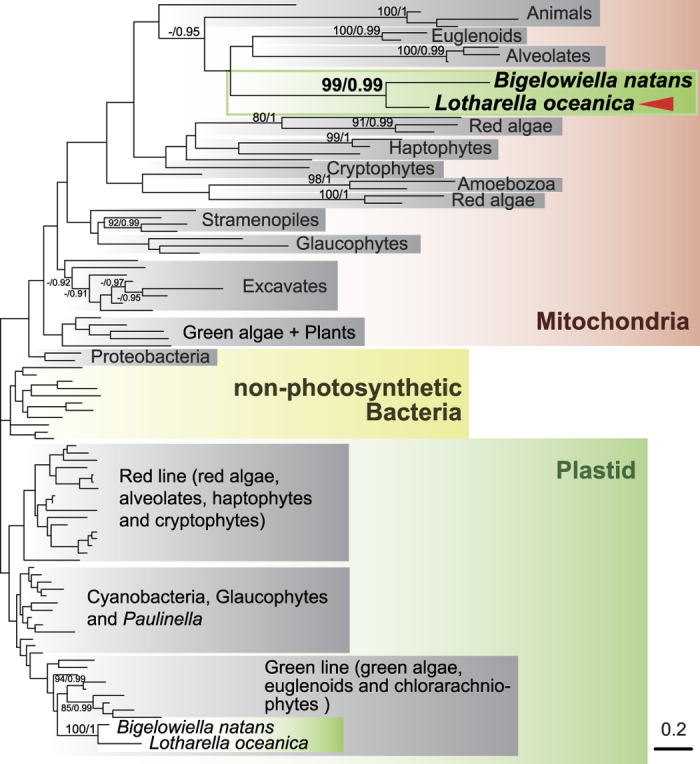
Maximum likelihood tree inferred from rpl16 protein sequences. Numbers on branches are ML bootstrap support percentages (left) and Bayesian posterior probabilities (right). No numbers are shown where bootstrap support was less than 80% or posterior probabilities were less than 0.9. Arrowhead indicates mitochondrial rpl16 protein sequence from the *Lotharella oceanica* nuclear genome. The scale bar shows the inferred number of amino acid substitutions per site.

In order to shed further light on the origins of the nucleus-encoded rps4 and rpl16 candidates in *L. oceanica*, phylogenetic analyses were performed. The rps4 candidate retrieved from the *L. oceanica* nuclear genome was only a fragment and showed significant similarity (e-value = 3e-13) only to the *B. natans* mitochondrial rps4 in blastp searches against the NCBI non-redundant (nr) protein database (the e-value of the second hit was 2.2). The rps4 gene could thus not be analyzed further. In the case of rpl16, 118 sequences from diverse taxa were retrieved from NCBI, aligned, and used for the inference of maximum likelihood and Bayesian trees (Fig. 3). Three major clades consisting of mitochondria + proteobacteria, plastids + cyanobacteria and non-photosynthetic bacteria (except proteobacteria) were resolved. Statistical support for these major clades was weak, as is typical of global phylogenetic trees inferred from a single protein gene, and cyanobacterial sequences were nested within plastid homologs (and not vice versa). Nevertheless, the *L. oceanica* rpl16 protein found in the nuclear genome branched within the mitochondrial rpl16 clade, and was monophyletic with the *B. natans* mitochondrial rpl16 protein with 99% and 0.99 support values. These results, together with the existence of a predicted mitochondrial targeting signal on the *L. oceanica* rpl16 protein, suggest that the rpl16 gene is indeed derived from mtDNA, and that its protein product is targeted to the mitochondrion.

Curtis *et al.* (2012) identified seven fragments of mtDNA in the *B. natans* nuclear genome but could find no examples of recent DNA transfers from the plastid and nucleomorph genomes^13^. Another study showed that in *B. natans* a spliceosomal intron in a nuclear GTPase superfamily gene is derived from a fragment of mtDNA^14^. In our gene-by-gene comparison, while *L. oceanica* and *B. natans* share the same plastid protein gene set, some gene content variation is observed in their nucleomorph genomes. However, ‘replacement’ nuclear genes for the missing nucleomorph protein genes (genes which are present in the *L. oceanica* or *B. natans* nucleomorph genomes) were not found based on analysis of genomic or transcriptomic data^21^. It is still under discussion how biological functions are maintained with fewer proteins in PPC^35^,^36^. In addition to these examples, the mitochondrial rpl16 EGT demonstrated herein is the first clear observation of a ‘recent’ EGT of a chlorarachniophyte organelle gene, recent in the sense that it occurred after the divergence of *Lotharella* and *Bigelowiella*. This further supports the idea that DNA transfer from mtDNA to the nuclear genome is more frequent than EGT of plastid and nucleomorph DNA, as suggested by Curtis *et al.* (2012)^13^.

### Rearrangement and duplication in chlorarachniophyte mitochondrial genomes

In order to assess the evolutionary dynamics of chlorarachniophyte mitochondrial genomes, the order of genes in the *Lotharella oceanica* and *Bigelowiella natans* genomes were compared. Unexpectedly, only five regions were found to have protein genes in the same order: nad3-nad7, atp6-nad4 L, rps11-rpl14, rps12-rps14 and nad9-nad6. The synteny of four RNA coding regions is also preserved, i.e., the rRNA operon, TrnP-TrnS, TrnW-Trnk and TrnC-TrnM.

Chlorarachniophyte mtDNAs have also been impacted by gene duplication. Duplicate nad6 and nad9 gene pairs are found adjacent to one another in two regions of *L. oceanica* mtDNA, separated by two tRNA genes (TrnA and TrnH) (Fig. 2). The nad6 and nad9 genes are single-copy in *B. natans*. Moreover, partial sequences of the nad7 and coxI genes were detected at positions 2566–2634 and 35876–36055 in *L. oceanica* mtDNA, respectively, in addition to the full-length homologs residing elsewhere. Although the duplicated regions are short, they are very similar to the intact nad7 and coxI genes (97% and 96% identity, respectively). It seems reasonable to speculate that the duplication and recombination events seen in the chlorarachniophyte mtDNAs examined here are related to the presence of repeat sequences in the genome (Fig. 1A).

**Figure 4:**
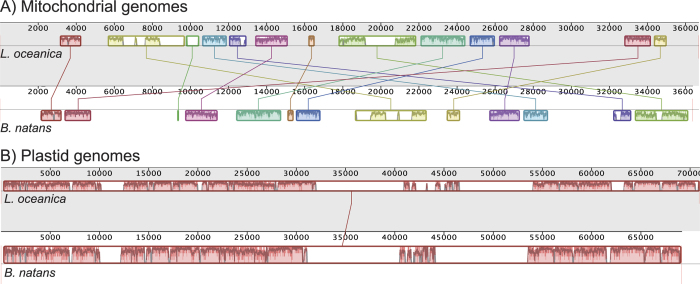
Chlorarachniophyte mitochondrial and plastid genome synteny. Images were generated using the Mauve genome alignment tool with default settings (Darling *et al.* 2004). (**A**) Mitochondrial genome synteny. (**B**) Plastidy genome synteny. Upper and lower lines of the genomes correspond to *Lotharella oceanica* and *Bigelowiella natans* respectively. Color-coded syntenic blocks indicate conserved segments (LCBs; Locally Collinear Blocks) identified by Mauve (minimum LCB weight = 339). Plots of sequence similarity are shown within each syntenic block. Regions with no color indicate no detectable homology between the two genomes with the settings used in MAUVE.

To assess the extent of mitochondrial genome rearrangements more closely, mitochondrial genomes were aligned using the Mauve genome aligner version 2.4.0^37^. A genome synteny map is shown in Fig. 4A. Thirteen syntenic regions, which are free from obvious genome rearrangement, are apparent (Fig. 4A). This result suggests at least 12 instances of mitochondrial genome rearrangements since *Lotharella* and *Bigelowiella* diverged from one another. The significant structural differences between *the L. oceanica* and *B. natans* mitochondrial genomes are especially interesting when compared to the plastid genomes of these two organisms. Unlike their scrambled mtDNAs, gene order in the chlorarachniophyte plastid genomes that have been sequenced is nearly identical^21^, yielding a single syntenic segment in a genome alignment (Fig. 4B). The nucleomorph genomes of *L. oceanica* and *B. natans* have also undergone frequent recombination since the two organisms diverged from one another (e.g., Tanifuji *et al.* 2014)^21^. What are the possible reasons for these differences?

### Organelle genome evolution in nucleomorph-bearing organisms

Table 3 summarizes the salient features of the three organellar genomes in nucleomorph-bearing algae. In the case of chlorarachniophytes, the plastid genome appears to be the most conservative, with high levels of synteny and similar gene complements between genera, as well as the apparent absence of NUPTs (in *Bigelowiella natans* at least)^13^,^21^. In chlorarachniophyte nucleomorph genomes, frequent rearrangements can be inferred based on the low level of synteny^19^,^21^. Intriguingly, some variation in the diversity of protein genes is found in the nucleomorph genomes of these algae (presumably due to gene loss)^16^,^19^,^21^ (Table 3). However, evidence for recent nucleomorph-to-host-nucleus EGT has not been found, even at the level of short DNA fragments (i.e., NUNMs)^13^,^35^. These observations are in stark contrast to the pattern seen in chlorarachniophyte mtDNAs, where genome rearrangements, EGTs and NUMTs are the norm. It is also noteworthy that based on pulsed-field gel electrophoresis, *Chlorarachnion reptans* appears to contain an unusually large mtDNA (~180 kbp in size) compared with the other chlorarachniophyte strains^30^ (Table 3).

Overall, it appears that chlorarachniophyte mtDNAs are changing rapidly, their nucleomorph genomes are experiencing rearrangement and gene loss, and their plastid genomes are highly conservative. Interestingly, this general trend parallels that seen in the other nucleomorph-bearing lineage, the cryptophytes, despite the different evolutionary origins of their host and endosymbiont^8^,^15^,^17^,^18^,^20^. Why? From the perspective of genome architecture, which factors correlate with organelle genome dynamics? The most fundamental difference between these three organelle genomes is that while mitochondrial and plastid genomes are of bacterial ancestry, nucleomorph genomes are eukaryotic. Prokaryotic and eukaryotic genomes are generally circular and linear, respectively, although numerous exceptions are known. In cnidarians, for example, the mitochondrial genome is linear and NUMTs are abundant, consistent with the hypothesis that EGT is more frequent in organisms with linear organellar genomes^38^. However, this is apparently not the case in nucleomorph-bearing organisms where the mtDNA of chlorarachniophytes is linear and the cryptophyte mtDNA is circular: NUMTs are found in both organisms but NUPTs and NUNMs are not. Therefore, the observed differences in organellar genome dynamics in chlorarachniophytes and cryptophytes do not obviously correlate with differences in genome structure.

Another difference between the mitochondria, plastids and nucleomorphs of chlorarachniophytes and cryptophytes is the number of organelles per cell. Where investigated, there is typically only one plastid and nucleomorph per cell, occasionally a few, in contrast to the presence of multiple mitochondria (usually ~10)^39^,^40^,^41^,^42^. It is noteworthy that the chlorarachniophyte *B. natans* and the cryptophyte *Guillardia theta*, whose nuclear genomes have been sequenced, each contain a single plastid and nucleomorph per cell but multiple mitochondria. Curtis *et al.* (2012) suggested that the presence of NUMTs and absence of NUPTs and NUNMs in their nuclear genomes could thus be explained by the “limited window transfer” hypothesis^13^,^43^. i.e., the frequency of EGT within a lineage is related to organelle abundance. Assuming that organelle lysis is the main source of DNA for EGT, the hypothesis predicts that the fewer organelles present in a cell, the less frequently viable opportunities to donate DNA to the nuclear genome arise. This hypothesis is supported by analysis of poly- and mono-organellar species across a wide range of plants and algae^44^. Our discovery of ‘recent’ EGT of mitochondrial rpl16 in *L. oceanica* is consistent with this idea.

The observed difference in genome arrangements between mtDNA and plastid genomes is controversial. Differences in genome stability through inter-molecular homologous recombination could be an explanation. Strauss *et al.* (1988) hypothesized that the large inverted repeat serves to stabilize plastid genomes, buffering them against rearrangement via inter-molecular recombination^45^. This possibility has been discussed elsewhere (e.g., Vieira *et al.* (2014) and Wei *et al.* (2015))^46^,^47^. Consistent with this idea, where investigated, the plastid genomes of chlorarachniophytes and photosynthetic cryptophytes maintain a large inverted repeat, while their mitochondrial genomes do not. In addition, the non-photosynthetic cryptomonad *C. paramecium* has lost one side of its plastid genome inverted repeat, and rearrangements are apparent when the genome is compared to those of other cryptophytes^22^,^25^. These observations are consistent with the hypothesis of Strauss *et al.* (1988).

Genomic copy number could also be a factor. All things being equal, an organelle with a low copy number is more likely to have genomic changes (e.g., rearrangements) fixed within the organelle. Indeed, in *B. natans* (chlorarachniophytes) and *G. theta* (cryptophytes), the genome was estimated to be present at 100–200 copies in each solitary plastid per cell, far higher than the genomic copy number for their multiple mitochondria (20–40 mtDNA copies per cell, or 2–4 copies per mitochondrion)^39^,^40^,^41^,^42^,^48^.

In sum, one explanation for the different evolutionary trends of organellar genomes in nucleomorph-bearing organisms, including EGT and genome rearrangements, is variation in the number of organelles per cell, the presence/absence of large inverted repeats, and genome copy number per organelle. The extent to which these factors play a role in organelle genome evolution beyond nucleomorph-bearing algae is unclear. The evolutionary distance between organisms being compared is a confounding factor, and the same tendencies may or may not be seen once divergence times have been controlled for. Of particular note, the primary plastids of green and red algae are much older than those of ‘secondary’ algae, and so the actual number of rearrangements and gene transfers that have occurred are difficult to discern. Nevertheless, useful information can still be obtained from comparing and contrasting patterns of organelle genome evolution in chlorarachniphytes and cryptomonads with the full range of plastid and/or mitochondrion-bearing eukaryotes in the future.

## Materials and Methods

### Sequencing and Assembling

Genome sequence data from *Lotharella oceanica* (CCMP622) were obtained as described by Tanifuji *et al.* (2014)^21^. Briefly, total cellular DNA was extracted and fractionated by cesium-chloride density gradient centrifugation, and an organelle genome-enriched fraction containing nucleomorph, plastid and mitochondrial (mtDNA) was purified. TruSeq library construction and Illumina HiSeq2000 sequencing were done at the Genome Quebec Innovation Center (Montreal, Quebec, Canada). The transcriptome data analyzed herein were generated at the National Center for Genome Resources (Santa Fe, NM, USA) as part of the Marine Microbial Eukaryote Transcriptome Project^49^. The RNA-seq data were deposited in the CAMERA portal under the project ID MMETSP0040.

The original genomic dataset containing 424,477,680 paired-end reads (100 base read lengths) was trimmed down to 95-base reads. Individual sequence reads in which >80% of the bases had quality scores of >20 were extracted using FASTAX-Tool kit (Ver. 0.0.13) (http://hannonlab.cshl.edu/fastx_toolkit/). Nucleomorph and plastid genome reads were removed using BWA mapping software (Burrows-Wheeler Aligner ver. 0.6.2)^50^ and in-house perl scripts. The remaining 9,381,044 paired-end and 13,290,186 single reads were assembled using three different methods. Two assemblies were generated using Ray (Ver. 2.1.0)^51^ with starting kmer sizes of 21 and 51. The resulting contigs were subjected to scaffolding procedures using SSPACE^52^ with the ‘extension’ option. A third set of scaffolds was generated using SPAdes (Ver. 3.0.0) with default settings^53^. The genome coverage depths of each scaffold were calculated according to Tanifuji *et al.* (2014)^35^. To identify mtDNA scaffolds in *L. oceanica*, tblastx searches were done using *Bigelowiella natans* mtDNA proteins (Accession HQ840955) as queries against the three different assemblies. Two mtDNA scaffolds with high coverage depths (~800×) were identified in each assembly, and preliminary investigation revealed that the predicted protein gene repertoires from the different assemblies were identical. However, the mtDNA sequences varied due to artificially duplicated regions. To correct inconsistencies between the different assemblies and to fill the gaps between mtDNA scaffolds, 21 PCR primers were designed. PCR amplifications were done using KOD FX Neo DNA polymerase (TOYOBO) and PCR products were cloned into Zero Blunt PCR Cloning Kit (Invitrogen). Individual clones were sequenced using an ABI 3100 sequencer (ABI) and Big Dye Ver. 3.1 (ABI).

### Gene annotation

ORFs greater than 90 amino acids in the *Lotharella oceanica* mtDNA were identified using Artemis (Ver. 16.0)^54^. Blastp searches were done for all predicted ORFs against the NCBI non-redundant (nr) protein database. Protein-coding regions were manually adjusted as necessary based on blast search alignments. Transfer RNA, rRNA and other components such as introns were searched for using RNAweasel (http://megasun.bch.umontreal.ca/cgi-bin/RNAweasel) with the genetic code setting of ‘Mold, Protozoan, and Coelenterate Mitochondrial; Mycoplasma/Spiroplasma’.

For mitochondrial rpl16 and rps4 genes, tblastn searches against *L. oceanica* transcriptome data were done using *B. natans* homologs. Top hit transcripts were used as queries in blastn searches against genomic scaffolds and to identify the corresponding protein-coding regions from *L. oceanica* scaffolds with low coverage depth. The precise rpl16 and rps4 coding regions from the *L. oceanica* genome were predicted using blast output alignments as guides. The *L. oceanica* mtDNA sequence has been deposited in GenBank under accession number KT806043.

### Southern hybridization analysis

Total genomic DNA from *Lotharella oceanica* was prepared using a standard cetyltrimethylammonium bromide (CTAB) protocol. Southern hybridation probes targeting the left and right ends of the *L. oceanica* mtDNA were amplified using the following primers: LoceMito_Left_spec_F (5′-AAGCAAAACGCAAGCAGAGG-3′), LoceMito_Left_spec_R (5′-GTGCGTTTTTGTAGGCCGTT-3′), LoceMito_Right_spec_F (5′-CGGATTCACCAACTCGTCAA-3′), and LoceMito_Right_spec_R (5′-TTTAGATGCTTCATCGCGCT-3′). PCR products were cloned into pGEM T-easy vector (Promega) and used as template for DIG (digoxygenin)-labeled probe synthesis according to the manufacture’s instructions in the PCR DIG labeling kit (Roche Diagnostics). One μg aliquots of total DNA were digested with *Hin*dIII, *Xho*I and *Acc*I restriction enzymes (Takara) at 37 °C overnight. After electrophoresis, digested DNA fragments were transferred to a positively charged nylon membrane by capillary transfer. Southern hybridization was carried out in DIG Easy Hyb buffer (Roche Diagnostics) with 2 μl/ml concentration of DIG-labeled probe at 37 °C overnight. The membranes were rinsed twice for 10 min at room temperature in low stringency buffer (0.1% SDS, 2 × SSC), followed by two 15 min rinses at 65 °C in high stringency buffer (0.1% SDS, 0.5 × SSC). Hybridization signals were detected using standard procedures for the DIG detection kit (Roche Diagnostics).

### Phylogenetic analysis

Mitochondrial, plastid and bacterial rpl16 protein sequences were retrieved from public databases for a diversity of eukaryotes and bacteria. 98 protein sequences were aligned using MAFFT (Ver. 7.212) with the global-pair option^55^. After manual exclusion of ambiguously aligned positions, the final dataset was comprised of 118 amino acid positions. A maximum likelihood (ML) phylogenetic tree was inferred using IQ-TREE Ver. 1.2.2 with the LG substitution matrix and Gamma (4 site rate categories) + Invar model, which was estimated as the best-fit model by IQ-TREE^56^. Bootstrap values were calculated using the standard non-parametric bootstrap method with 100 replicates. Bayesian analyses using the CAT-LG + Γ model were carried out with the same alignment using PHYLOBAYES v3.3^57^. Markov chain Monte Carlo chains (MCMC) were run for 80,000 generations in two independent chains. Every two trees were collected with the initial 20,000 trees being discarded as burn-in. The chains for analyses converged with a maxdiff = 0.1. Subsequently, the consensus tree with branch lengths and Bayesian posterior probabilities (BPPs) was calculated from the rest of the sampled trees.

### Genome rearrangement comparison

The *Bigelowiella natans* mitochondrial genome (accession: HQ840955), *B. natans* plastid genome (HQ851108) and *Lotharella oceanica* plastid genome sequences (KF438023) were obtained from Genbank. Mitochondrial and plastid genomes were aligned separately using the Mauve genome aligner version 2.4.0^37^ with default settings.

## Additional Information

**How to cite this article**: Tanifuji, G. *et al.* Comparative genomics of mitochondria in chlorarachniophyte algae: endosymbiotic gene transfer and organellar genome dynamics. *Sci. Rep.* **6**, 21016; doi: 10.1038/srep21016 (2016).

## Supplementary Material

Supplementary Information

## Figures and Tables

**Table 1:** Summary of chlorarachniophyte mitochondrial genomes.

Strain	*L. oceanica*(CCMP622)	*B. natans*(CCMP2755)*
Genome Size (kbp)	36.7	36.4
# protein genes (# hypothetical protein genes)	35 (9)	34 (7)
# RNA genes		
rRNAs	3	3
tRNAs	24	26
Overall G + C content (protein coding regions)(%)	50.14 (49.22)	42.15 (40.81)
Gene density (genes/kbp)	0.95	0.93

^*^Data taken from Genbank (accession number HQ840955).

**Table 2:** *Lotharella oceanica* and *Bigelowiella natans* mitochondrial gene content.

Protein genes	*L. oceanica*	*B. natans*	RNA genes	*L. oceanica*	*B. natans*
*atp1*	•	•	*trnA*(tgc)	•	•
*atp6*	•	•	*trnC*(gca)	•	•
*atp8*	—	•	*trnD*(gtc)	•	•
*atp9*	•	•	*trnE*(ttc)	•	•
*cob*	•	•	*trnF*(gaa)	•	•
*cox1*	•	•	*trnG*(tcc)	—	•
*cox2*	•	•	*trnH*(gtg)	•	•
*cox3*	•	•	*trnI*(gat)	•	•
*nad1*	•	•	*trnK*(ttt)	•	•
*nad2*	•	•	*trnL*(caa)	•	•
*nad3*	•	•	*trnL*(taa)	•	•
*nad4*	•	•	*trnL*(tag)	•	•
*nad4L*	•	•	*trnM*(cat)	• (×3)	• (×3)
*nad5*	•	•	*trnN*(gtt)	•	•
*nad6*	• (×2)	•	*trnP*(tgg)	•	•
*nad7*	•	•	*trnQ*(ttg)	•	•
*nad9*	• (×2)	•	*trnR*(tcg)	—	•
*rpl5*	•	•	*trnR*(tct)	—	•
*rpl6*	•	•	*trnS*(gct)	•	•
*rpl14*	•	•	*trnS*(tga)	•	•
*rpl16*	—	•	*trnV*(tac)	—	•
*rps3*	•	•	*trnW*(cca)	•	•
*rps4*	—	•	*trnW*(tca)	• (×2)	•
*rps7*	•	•	*trnY*(gta)	• (× 2)	•
*rps11*	•	•			
*rps12*	•	•	LSU RNA	•	•
*rps14*	•	•	SSU RNA	•	•
hypothetical ORFs	9	7	5S RNA	•	•

**Table 3:** Overview of organelle genomes in nucleomorph-bearing organisms.

Genomes	Mitochondria		Nucleomorphs		Plastids	
Lineages	Chlorarachniophytes	Cryptophytes	Chlorarachniophytes	Cryptophytes	Chlorarachniophytes	Cryptophytes
Genome structure	Linear	Circular	Linear		Circular	
Genome size (kbp)	34–180	48–60	330–1,000	500–850	Ca. 70	120–150
Genomic copy number per cell	18–40	24–43	2	4	130–260	120–150
Recombination (based on synteny)	Rearranged		Rearranged		Conserved	
Recent organelle-to-nucleus DNA transfers (i.e., NUMTs, NUNMs and NUPTs)	Detected		Not detected		Not detected	
Loss of protein genes with predicted functions (# variable/# total genes)*	3/29	4/40	27/198	94/310	0/60	1/127**

Data taken from the present study and previous publications (Curtis *et al.* 2012; Douglas and Penny 1999; Douglas *et al.* 2001; Gilson and McFadden 1999; Gilson *et al.* 2006; Hauth *et al.* 2005; Hirakawa *et al.* 2014; Khan *et al.* 2007; Kim *et al.* 2008; Kim *et al.* 2015; Lane *et al.* 2006; Lane *et al.* 2007; Rogers *et al.* 2007; Silver *et al.* 2007; Suzuki *et al.* 2015; Tanifuji *et al.* 2010; Tanifuji *et al.* 2014a, 2014b; Moore *et al.* 2012). The plastid and nucleomorph genomes of the non-photosynthetic species *Cryptomonas paramecium* were not included in this table (Donaher *et al.* 2009; Tanifuji *et al.* 2011). *The ‘# total genes’ values indicate the total number of protein genes with predicted functions found in the completely sequenced genomes, while ‘# variable’ indicates the number of protein genes missing from at least one of the studied species. Proteins without obvious functions (e.g., hypothetical ORFs) and pseudogenes were not counted. **The laterally transferred DNA polymerase III (dnaX) gene was ignored.
