# Supplementary material for: Comparative genomics of mitochondria in chlorarachniophyte algae: endosymbiotic gene transfer and organellar genome dynamics
Source: Sci Rep. 2016 Feb 18;6:21016. doi: 10.1038/srep21016 (PMC4757882; doi:10.1038/srep21016)
Supplement: Supplementary Information [file srep21016-s1.pdf]

Comparative genomics of mitochondria in chlorarachniophyte algae: endosymbiotic gene transfer and organellar genome dynamics

Goro Tanifuji[1*], John M. Archibald[2,3], Tetsuo Hashimoto[1]

1, Faculty of Life and Environmental Sciences, University of Tsukuba (Japan)

2, Department of Biochemistry and Molecular Biology, Dalhousie University (Canada)

3, Program in Integrated Microbial Biodiversity, Canadian Institute for Advanced Research, Toronto, Ontario (Canada)

*Author for correspondence:

Goro Tanifuji

Email: tanifuji.goro.gn@u.tsukuba.ac.jp

Suppl. Fig. 1

A) Mitochondrial rpl16 genomic sequence

```
5`- cccaaccATGCCAAACCCGTGGCGCAAAATATTCCTACGGCGCCTCCTGCGGCCCAAGATCCCAAAG
            M  P  N  P  W  R  K  I  F  L  R  R  L  L  R  P  K  I  P  K

    GCTTCGACGACAAACAAGTCGCTACTCCTTGGCAAGTTTGGCGTCCTGAGTACTGAGGCAGGCCT
     A  S  T  T  N  K  S  L  L  L  G  K  F  G  V  L  S  T  E  A  G  L

    CCTCACAAATGCGCAGAAAGAAGCTGgtacctatcctatcagaaaattgagctccttcaccgtaaccgcgaccttgaagaatga
      L  T  N  A  Q  K  E  A

    tgtgttccattcgaacacgtgtgtgtgtgtgaccccgtatatatttgtgtgcttgaaggctcatgaaactgatgtgtttagTTCGACTGTCGATG
                                                                                      V  R  L  S  M

    GGGCGTAAACTGAAGCCGATGGGAGGGCGGTTTTGGATCCACATAAATCACTTTATACCGCTTAC
     G  R  K  L  K  P  M  G  G  R  F  W  I  H  I  N  H  F  I  P  L  T

    GCGTAAAGCCAAGGGCGCTAGGATGGGTAAGGGTAAGGGAGCGGCTCTTACTGGCGGGAAGGC
      R  K  A  K  G  A  R  M  G  K  G  K  G  A  A  L  T  G  G  K  A

    GCCGGTCAGACCCGGGACGGTTATAATAGAATTCAGCGGGGTTGATGAATTGGAGGCGAAGGCC
      P  V  R  P  G  T  V  I  I  E  F  S  G  V  D  E  L  E  A  K  A

    CTTCACTCGTCGATTACAAAGAAGCTTCCTGTGAAGACAAGGCTCATCACAAACAAATTCAACGAA
     L  H  S  S  I  T  K  K  L  P  V  K  T  R  L  I  T  N  K  F  N  E

    GCTATTGTTTAGattttagatg -3`
     A  I  V  +
```

B. Mitochondrial rps4 genomic sequence (partial)

```
5`- atcatcagGTCGGAAGTACAACGACTTCAAGAGGAAGCTCCCATGGGCGCGCTACCTCCGTTACTTCCT
             G  R  K  Y  N  D  F  K  R  K  L  P  W  A  R  Y  L  R  Y  F  L

    GTACCACAATCCAGgtgggatgtgaacgatgatcatcagaatgaggacgatggttgtgtatgaaaatggcgaggacgatgatgatggtg
      Y  H  N  P

    atgatgatgacgacgacaaccaacgacgatgacagcgtgataatgaagacgatgacgaatgatgatgacgacgatgacaagaatgatgacaa

    gaatgatgtcaagaatgatgatggcgaggatgatgatgatgagaaccacggtgatgatgagggtgcctaaaatgatgagcaaccgccatgcgcg

    agagtgacattgcgaaaacttgacgatggatccctcttggtagAATTCATCTGCAACTACAACACGCTCACTGCCATCTAC
                                                E  F  I  C  N  Y  N  T  L  T  A  I  Y

    CGCCCGATTAACACCACGGAGAGAGCTCACGGAGTGAGATACCCTCGCCACTTTGGCCCCGGGCT
     R  P  I  N  T  T  E  R  A  H  G  V  R  Y  P  R  H  F  G  P  G  L

    TTTCTTCTGGAGGGAGGTGTACAACTTCTATCGCTCTTAAgcgta -3`
      F  F  W  R  E  V  Y  N  F  Y  R  S  #
```

Supplementary Figure 1: Nuclear genomic sequences of transferred mitochondrial protein genes in *Lotharella oceanica*. A) Nuclear genomic sequence of the mitochondrial rpl16 gene. B) Partial nuclear genomic sequence of the mitochondrial rps4 protein gene. Capital letters and lower case letters correspond to protein coding regions and non-coding regions, respectively. Predicted amino acid sequences are shown below the nucleotide sequences.
